# Effect of boswellia (*Boswellia serrata L.*) supplementation on glycemic markers and lipid profile in type 2 diabetic patients: a systematic review and meta-analysis

**DOI:** 10.3389/fcdhc.2024.1466408

**Published:** 2024-10-10

**Authors:** Mehdi Karimi, Kimia Vakili, Pegah Rashidian, Seyedeh-Kiana Razavi-Amoli, Matin Akhbari, Kimia Kazemi

**Affiliations:** ^1^ Faculty of Medicine, Bogomolets National Medical University (NMU), Kyiv, Ukraine; ^2^ Systematic Review and Meta-analysis Expert Group (SRMEG), Universal Scientific Education and Research Network (USERN), Tehran, Iran; ^3^ Student Research Committee, School of Medicine, Shahid Beheshti University of Medical Sciences, Tehran, Iran; ^4^ Reproductive Health Research Center, School of Medicine, Guilan University of Medical Sciences (GUMS), Rasht, Iran; ^5^ Student Research Committee, School of Medicine, Mazandaran University of Medical Sciences (MazUMS), Sari, Iran; ^6^ Faculty of Medicine, Istanbul Yeni Yuzyil University, Istanbul, Türkiye; ^7^ Department of Food Science and Technology, Ayatollah Amoli Branch, Islamic Azad University, Amol, Iran

**Keywords:** boswellia, Kundur, herb, diabetes, anti-diabetic, glycemic markers, lipid profile, meta-analysis

## Abstract

**Background:**

Type 2 diabetes mellitus (T2DM) is a significant global health challenge whose prevalence is projected to increase alarmingly. Recently, due to better safety and fewer adverse effects, herbal medicines have been used to manage T2DM. This study aimed to evaluate the efficacy of boswellia in improving glycemic markers and lipid profiles in T2DM patients.

**Methods:**

A comprehensive search was conducted on the PubMed, Web of Science, and Scopus databases for all relevant studies published up to April 30, 2024. The effects of boswellia supplementation were evaluated using glycemic markers and lipid profiles. The data were extracted and meta-analyzed using Stata software.

**Results:**

This meta-analysis included five studies with a total of 287 patients with T2DM. It was found that boswellia in patients with T2DM compared to the placebo or control group significantly reduced hemoglobin A1C (HbA1C) (SMD: -1.01; 95%CI: -1.55 to -0.46; P=0.00), total cholesterol (TC) (SMD: -0.44; 95%CI: -0.68 to -0.21; P=0.00), Triglycerides (TG) (SMD: -0.42; 95%CI: -0.66 to -0.19); P=0.00) and low-density lipoprotein (LDL) (SMD: -0.43; 95%CI: -0.73 to -0.12); P=0.006) levels, while reduced fasting blood glucose (FBG) but it was not significant (SMD: -1.34, 95%CI: -2.68 to 0.00; P=0.05). Notably, it did not affect high-density lipoprotein (HDL) (SMD: 0.56, 95%CI: -0.14 to -1.26; P=0.118).

**Conclusion:**

In summary, boswellia supplementation has the potential to improve glycemic markers and lipid profiles in patients with T2DM. It may help diabetic patients in addition to a controlled diet and other treatments.

**Systematic review registration:**

crd.york.ac.uk/PROSPERO/display_record.php?RecordID=538347, identifier CRD42024538347.

## 1 Introduction

Type 2 Diabetes Mellitus (T2DM) is a prevalent chronic endocrine and metabolic disorder globally, characterized by long-term hyperglycemia, insulin resistance, and relative insulin insufficiency (1). The 2021 International Diabetes Federation report reported 536.6 million people diagnosed with T2DM (2). Diabetes prevalence is projected to rise to 642.7 million by 2030 and 783.2 million globally by 2040, with one in two adults unaware of their condition. Modifying risk factors like obesity, smoking, hypertension, dyslipidemia, and cardiovascular diseases can reduce the prevalence and incidence of diabetes, a common condition that increases the likelihood of developing the disease (3, 4) T2DM can also stimulate other diseases, such as cardiovascular diseases, making it a significant risk factor (5, 6).

Despite the widespread use of medications and lifestyle modifications among diabetic patients, many individuals turn to complementary and alternative medicine (CAM) and herbal remedies due to their affordability and potentially fewer side effects (7). In recent years, there has been growing interest in the use of herbal supplements as adjunct therapy for the management of T2DM (8–11). Notably, emerging evidence suggests that some herbals may exert a more comprehensive effect on both glycemic indicators and lipid profiles. For instance a study on okra reported a reduction in fasting blood glucose (FBG) without impacting glycated hemoglobin in T2DM patients (9), boswellia appears to offer broader benefits (12–16).

*Boswellia serrata* is a traditional medicinal plant used in Ayurveda since ancient times due to its anti-inflammatory and therapeutic properties. Its active components, particularly boswellia acids, have been reported to offer potential benefits by altering pathways leading to diabetes and related complications via inflammation (17–20). To assess the effectiveness of boswellia, several studies in animal populations investigated the anti-diabetic role of *Boswellia serrata* (8–10). Additionally, a few randomized clinical trials (RCT) evaluated the supplementation of *Boswellia serrata* extracts in human patients with T2DM and assessed its effects on the glycemic markers and lipid profile (12–16).

The consistency of reported outcomes across studies on boswellia supplementation in improving glycemic markers and lipid profiles in patients with type 2 diabetes remains unclear. A comprehensive evaluation of available evidence is needed to determine its efficacy and safety. This study assesses the effectiveness of boswellia on glycemic indicators and lipid profiles in T2DM patients, aiming to better understand its potential role as a complementary treatment option. This will inform clinical practice and guide future research in this area.

## 2 Materials and methods

This systematic review and meta-analysis was conducted based on the PRISMA (Preferred Reporting Items for Systematic Reviews and Meta-Analyses) statements (21). The methodology encompasses critical steps to ensure transparency and rigor in the research. The review protocol was registered in the International Prospective Register of Systematic Reviews (PROSPERO) under registration #CRD42024538347.

### 2.1 Search strategy

We performed a comprehensive systematic search across four online databases: PubMed, Scopus, Web of Science, and Embase, covering publications from their inception through April 30, 2024. Additionally, we manually searched Google Scholar to identify relevant gray literature. Our search strategy involved an exhaustive examination of Medical Subject Headings (MeSH) terms in the title and abstract ([title/abstract]) for Boswellia, glycemic markers, and lipid profile.

Our research question was formulated using the Patient, Intervention, Comparison, and Outcome (PICO) framework, which guided the selection of studies and ensured the relevance and specificity of the search strategy (**Table 1**) (22).

**Table 1:** The population, intervention, comparison, outcome, study design (PICO) criteria.

Domain	Criteria Selection
Participants	Individuals of any age with Type 2 diabetic melilites
Intervention group	Supplementation of Boswellia (Kundur) or Boswellia extract
Comparison group	Control, Placebo, no treatment,
Outcomes	Changes in lipid profile and glycemic markers
Query words ([Title/Abstract])	Boswellia OR Kundur OR Aflapin OR Frankincense OR olibanum OR Shallaki AND Diabetes OR Diabetic OR Prediabetic OR “Type 2 diabetes” OR glycemic OR hyperglycemic OR hyperglycemia OR “blood glucose” OR “blood sugar” OR “plasma glucose” OR “glucose tolerance” OR HbA1C OR “hemoglobin A1C” OR “glycohemoglobin*” OR “glycated hemoglobin” OR insulin OR “lipid profile” OR “blood lipid” OR “plasma lipid” OR “blood fat” OR lipoprotein OR Cholesterol OR Triglyceride* OR Triacylglycerol* OR HDL OR “high-density lipoprotein” OR LDL OR “low-density lipoprotein”

### 2.2 Inclusion and exclusion criteria

Studies were included if they involved individuals with T2DM of any age and health status, with interventions consisting of boswellia or its extract and comparisons to placebo, no treatment, or alternative interventions, specifically examining changes in lipid profile and glycemic markers. Exclusion criteria encompassed studies involving participants with other types of diabetes (e.g., type 1 diabetes mellitus, gestational diabetes), records including pregnant or breastfeeding women, non-human studies (animal, *in vitro*, and *in vivo* studies), non-English language publications, and reviews, editorials, and opinion pieces.

### 2.3 Study selection

Two independent reviewers (P.R. & M.A.) conducted a multi-step screening process for the identified studies to ensure the inclusion of relevant and high-quality data. Initially, they performed a title and abstract review to eliminate studies that did not meet the predefined criteria quickly. Studies deemed potentially relevant underwent a detailed full-text assessment, where the reviewers evaluated them against specific inclusion and exclusion criteria, such as study design, population characteristics, interventions, and outcomes. Any discrepancies between reviewers were resolved through discussion, and if consensus was not reached, a third reviewer (M.K.) provided an independent evaluation. Throughout the selection process, the reasons for excluding studies were documented to ensure transparency and reproducibility.

The screening process involved three steps: removing duplicates with reference management software, reviewing titles, abstracts, and keywords, followed by full-text assessment based on PICO criteria, and finally, selecting relevant studies for analysis.

### 2.4 Data extraction

Two individual reviewers (P.R. and M.A.) independently performed data extraction, ensuring accuracy and consistency through a cross-over verification process. In cases of disagreement or conflict, resolution was achieved through discussion and consultation with a third reviewer (M.K.), followed by double-checking the extracted data to confirm accuracy. Data from each included article were systematically compiled across five key categories: general information (such as the first author, publication year, country of origin, and study design), population characteristics (age and gender), intervention features (form, dose, and duration of boswellia administration), comparative analysis (control groups including placebo, no treatment, or alternative interventions), and primary outcomes (glycemic markers and lipid profile). This rigorous and comprehensive approach assured accurate capture and analysis of all relevant data, providing a robust basis for evaluating the effects of boswellia on specified health outcomes.

### 2.5 Interpretation and recommendations

The results were interpreted in the context of existing literature, highlighting the clinical implications of boswellia on lipid profile and glycemic markers. Recommendations for future research were proposed based on the gaps identified in the current evidence.

### 2.6 Risk of bias assessment

Two researchers (M.K. & K.V.) evaluated the methodological quality of each included study through the Revised Cochrane risk-of-bias tool for randomized trials (RoB 2). This tool was built to assess the risk of bias in randomized clinical trial studies (23). The tool consists of 5 items that concern five domains, including selection bias, reporting bias, performance bias, attrition bias, and other sources of bias.

### 2.7 Meta-analysis

We used Stata version 18 (Stata Corp, College Station, TX, USA) software to calculate the pooled standard mean difference (SMD) [95% confidence interval (CI)] of serum lipid profile levels in diabetic patients receiving boswellia treatments versus those who received placebo or no treatment in both pretreatment and posttreatment. We also performed SMD analysis to compare the pretreatment blood levels of lipid profiles to the posttreatment levels in both groups. In addition, we used the inconsistency index (I**^2^**) and Cochrane Q to examine the heterogeneity of the included studies. An I² greater than 50% or a p-value less than 0.05 indicated significant heterogeneity among the studies. Pooled prevalences were estimated using a random-effect model. In addition, we assess the effect of potential factors. The confidence intervals for I² were reported to determine the degree of heterogeneity among the studies (24).

## 3 Results

### 3.1 Study selection

**Figure 1:**
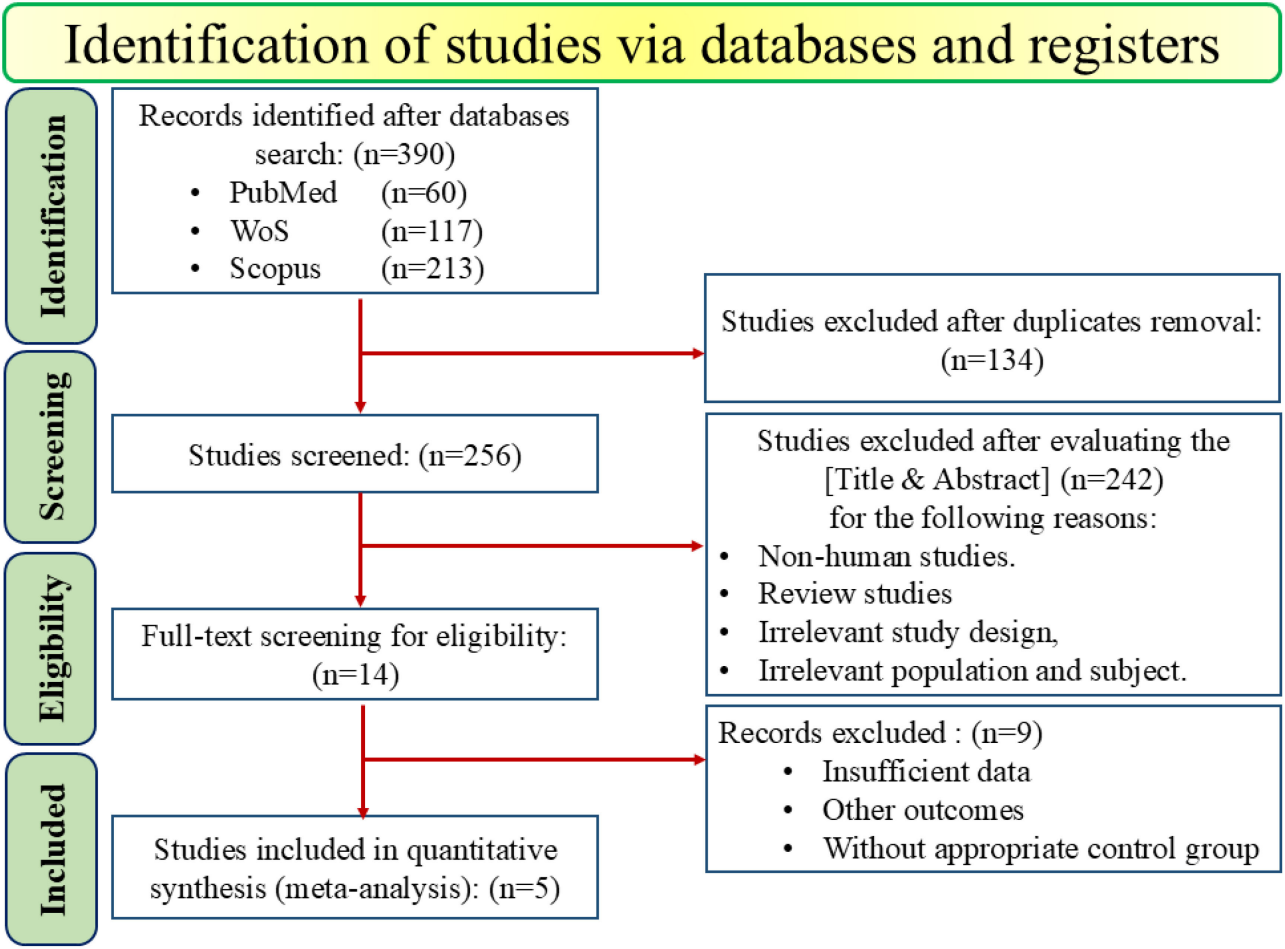
PRISMA flow diagram of study selection for inclusion trials in the systematic review.

A comprehensive systematic search across online databases, including PubMed (n=60), Web-of-Science (n=117), and Scopus (n=213), initially identified 390 studies. Also, to find any gray literature, Google Scholar was manually searched. After removing 134 duplicates, 256 studies were screened based on the [Titles and Abstract]. During this screening process, 242 additional records were excluded due to non-human studies (*in vivo*, *in vitro*, etc.), review studies, irrelevant studies, and population. This left 14 studies for full-text review to assess their eligibility. Of these, 9 studies were excluded primarily due to insufficient data, other outcomes, or without appropriate treatment or control groups. Finally, 5 papers published between 2014 and 2024 were included in the meta-analysis, as shown in **Figure 1**.

### 3.2 Study characteristics and demographic data

The five studies included in the meta-analysis involved 287 patients with T2DM. A total of 144 individuals received the boswellia supplementation, and 143 patients in the control group received either a placebo or no treatment (in 3 of 5 studies, subjects received a placebo; in the other 2, they received nothing). The dosage regimen for the boswellia supplementation ranged from 500 to 1200 mg per day, with treatment durations spanning 6, 8, or 12 weeks. The form of Boswellia, in one study, was powder (16), and in the remaining 4 studies were resin (12–15) (**Table 2**).

**Table 2:** General characteristics of included studies in the meta-analysis.

Author/Year	Country	Study design	Population	Sample Size (Cont/Int)	Age	Gender	Intervention/Dose	Dose	Duration	Outcomes
Kahodom Mohson et al., 2021 (16)	Iraq	Quasi-experimental non-randomized	T2DM	40 (20/20)	31–57	M:24 F:16	Boswellia powder 1200 mg/day	1200 (mg/day)	12 weeks	HbA1C, TC, TG, HDL. LDL,
Mehrzadi et al. 2018 (15)	Iran	RCT	T2DM	56 (29/27)	18–65	M:26 F:30	Boswellia gum resin 500 mg/day	500 (mg/day)	8 weeks	FBG, HbA1C, TC, TG, HDL. LDL,
Khalili et al. 2017 (14)	Iran	RCT	T2DM	60 (30/30)	40–60	M:30 F:30	Boswellia gum resin 600 mg/day	600 (mg/day)	12 weeks	FBG, HbA1C,
Ahangarpour et al. 2014 (12)	Iran	RCT	T2DM	60 (30/30)	30–48	M:30 F:30	Boswellia gum resin 900 mg/day	900 (mg/day)	6 weeks	TC, TG, HDL. LDL,
Azadmehra et al. 2014(13)	Iran	RCT	T2DM	71 (34/37)	18–65	M:42 F:29	Boswellia resin 800 mg/day	800 (mg/day)	12 weeks	FBG, HbA1C, TC, TG. LDL,

Cont, control; Int, intervention; RCT, Randomized controlled trial; T2DM, Type 2 Diabetes mellitus; M, male; F, female.

The study population was pretty balanced in terms of gender, with 52.32% (95%CI: 41.26-63.27; I**^2^ =** 26.98%) of cases and 55.04% (95%CI: 45.03-64.86; I**^2^ =** 10.48%) of controls being men. The age range of all studies varied from 18 to 65 years, with most patients falling in their 30-50th decades of life. In the intervention group, the mean age and BMI were 54.57 (95%CI: 51.34-57.80; I**^2^ =** 52.9%) and 27.01 (95%CI: 24.93 – 29.09; I**^2^ =** 78.6%), respectively. Controls’ pooled mean age and BMI were 53.66 (95%CI: 50.2 -57.30; I**^2^ =** 56.6%) and 26.58 (95%CI: 23.77 -29.40; I**^2^ =** 81.1%), respectively. The mean BMI between the two groups showed no significant difference (SMD=0.17; 95%CI: -0.12 to 0.46; I**^2^ =** 0.0%, P=0.251) (**Table 3**).

**Table 3:** Meta-analysis of included studies.

Data	Variable	Number of studies	Group		ES (95% CI)	I^2^ (%)	P-Value
**Demographic data**	Male (%)	4	Control		55.04 [45.03, 64.86]	10.5	–
			Intervention		52.32 [41.26, 63.27]	26.9	–
	Age (mean)	3	Control		53.66 [50.02, 57.30]	56.6	–
			Intervention		54.57 [51.34, 57.80]	52.9	–
	BMI (mean)	3	Control		26.58 [23.77, 29.40]	81.1	–
			Intervention		27.01 [24.93, 26.09]	78.6	–
	BMI (SMD)	3	–		0.17 [-0.12, 0.46]	0.0	0.251
**Lab data**	FBG (SMD)	3	Intervention vs. Control	Pre-treatment	0.05 [-0.23, 0.34]	0.0	0.711
				Post-treatment	-1.34 [-2.76, 0.08]	94.6	0.065
			Pre-treatment vs. Post-treatment	Control	-0.12 [-0.41, 0.16]	0.0	0.396
				Intervention	-1.34 [-2.68, 0.00]	94.0	0.049 = 0.05
	HbA1C (SMD)	4	Intervention vs. Control	Pre-treatment	-0.14 [-0.40, 0.12]	0.0	0.288
				Post-treatment	-1.18 [-1.83, -0.54]	79.8	0.000
			Pre-treatment Vs. Post-treatment	Control	-0.19 [-0.45, 0.07)	0.0	0.159
				Intervention	-1.01 [-1.55, -0.46]	73.5	0.000
	TC (SMD)	5	Intervention vs. Control	Pre-treatment	-0.05 [-0.37, 0.27]	46.7	0.746
				Post-treatment	-0.59 [-1.20, 0.03]	84.1	0.060
			Pre-treatment vs. Post-treatment	Control	0.01 [-0.22, 0.24]	0.0	0.930
				Intervention	-0.44 [-0.68, -0.21]	0.0	0.000
	TG (SMD)	5	Intervention vs. Control	Pre-treatment	-0.02 [-0.27, 0.22]	10.0	0.854
				Post-treatment	-0.39 [-0.62, -0.15]	0.0	0.001
			Pre-treatment vs. Post-treatment	Control	-0.07 [-0.30, 0.16]	0.0	0.541
				Intervention	-0.42 [-0.66, -0.19]	0.0	0.000
	HDL (SMD)	4	Intervention vs. Control	Pre-treatment	0.02 [-0.25, 0.28]	0.0	0.903
				Post-treatment	0.50 [-0.47, 1.47]	91.3	0.311
			Pre-treatment Vs. Post-treatment	Control	0.18 [-0.21, 0.56]	51.3	0.367
				Intervention	0.56 [-0.14, 1.26]	83.8	0.118
	LDL (SMD)	5	Intervention vs. Control	Pre-treatment	-0.06 [-0.56, 0.44]	70.2	0.805
				Post-treatment	-0.61 [-1.32, 0.11]	88.2	0.097
			Pre-treatment vs. Post-treatment	Control	0.03 [-0.20, 0.27]	0.0	0.775
				Intervention	-0.43 [-0.73, -0.12]	39.4	0.006

FBG, fasting blood glucose; HbA1C, hemoglobin A1C; TC, total cholesterol; TG, triglyceride; HDL, high-density lipoprotein; LDL, low-density lipoprotein; SMD, standard mean difference.

### 3.3 Quality assessment

Risk of bias assessment.

Our results showed that four studies had “Some concerns,” suggesting the potential for bias in certain domains, though not significant enough to fully compromise the findings. One study exhibited a “High risk of bias,” indicating serious issues that could affect the reliability of its results. More details regarding quality assessment are mentioned in **Table 4**.

**Table 4:** Risk of bias assessment (ROB-2).

Author/Year	D1	D2	D3	D4	D5	Overall
KahodomMohson et al. 2021 (16)	H	H	L	L	Some concerns	H
Mehrzadi et al. 2018 (15)	L	L	L	Some concerns	L	Some concerns
Khalili et al. 2017 (14)	L	L	L	Some concerns	L	Some concerns
Ahangarpour et al. 2014 (12)	L	Some concerns	L	Some concerns	Some concerns	Some concerns
Azadmehr et al. 2014 (13)	L	Some concern	L	Some concerns	L	Some concerns

✓D1: Bias arising from the randomization process.

✓D2: Bias due to deviations from intended interventions.

✓D3: Bias due to missing outcome data.

✓D4: Bias in measurement of the outcome.

✓D5: Bias in selection of the reported result.

✓L, low risk of bias; H, high risk of bias.

### 3.4 Effect of boswellia on FBG & HbA1C

**Figure 2:**
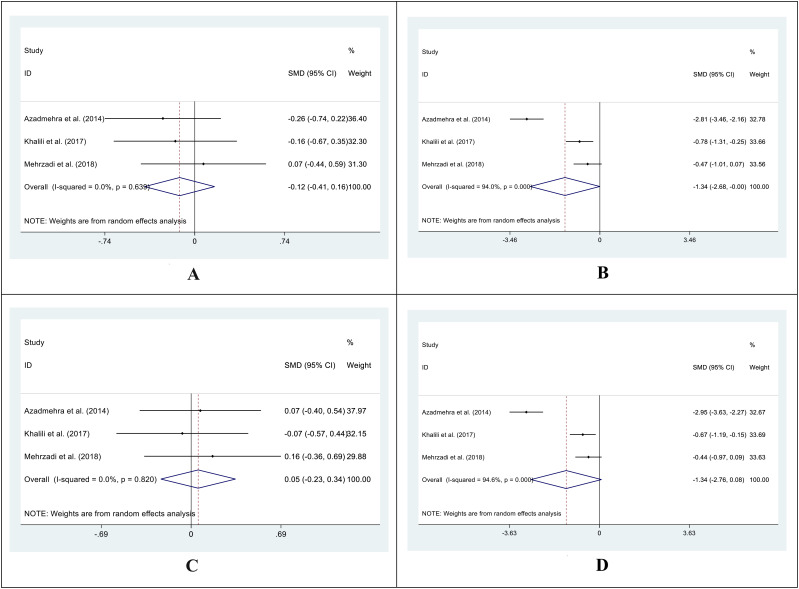
Forest plot of SMD for the FBG (**A**: before vs. after in the control groups; **B**: before vs. after in the intervention groups; **C**: intervention vs. control groups before supplementation; **D**: intervention vs. control groups after supplementation).

In 3 out of 5 studies, we retrieved the mean and SD of the fasting blood glucose (FBG) level in both intervention and control groups before and after the supplementation. The SMD of before and after supplementation levels of FBG in control and intervention groups was -0.12 (95%CI: -0.41 - 0.16; I^2^ = 0.0%; P=0.396) (**Figure 2A**) and -1.34 (95%CI: -2.68 - 0.00; I^2^ = 94.0%; P=0.049) respectively (**Figure 2B**). We also conducted an SMD analysis between the treatment and control groups in both time points and retrieved 0.05 (95%CI: -0.23 - 0.34; I^2^ = 0.0%; P=0.711) (**Figure 2C**) and -1.34 (95%CI: -2.76 - 0.08; I^2^ = 94.6%; P=0.065) (**Figure 2D**) for before and after supplementation levels, respectively.

**Figure 3:**
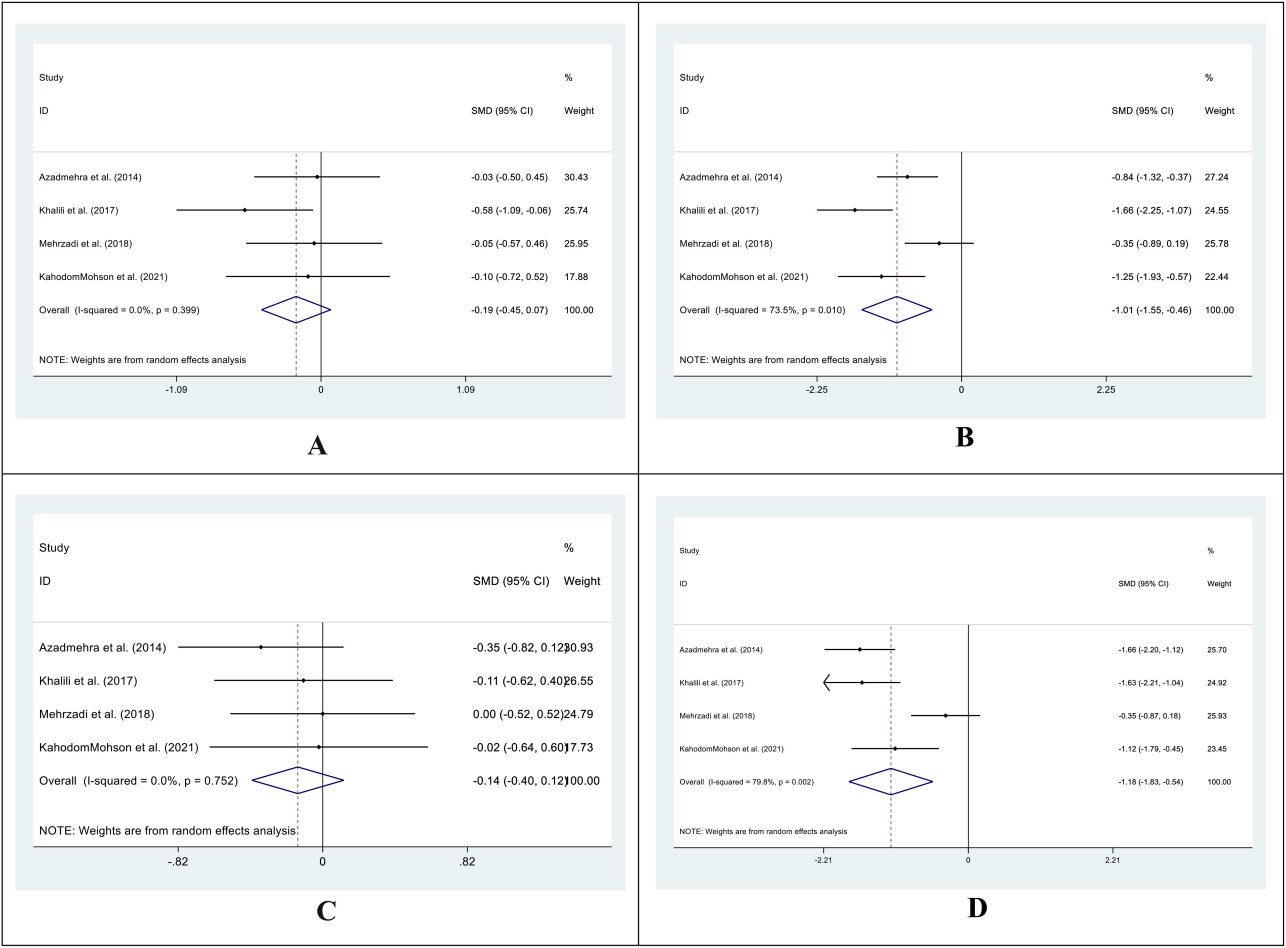
Forest plot of SMD for the HbA1C. (**A**: before vs. after in the control groups; **B**: before vs. after in the intervention groups; **C**: intervention vs. control groups before supplementation; **D**: intervention vs. control groups after supplementation).

In 4 out of 5 studies, we collected data on the mean and SD of HbA1C levels in both the intervention and control groups before and after supplementation. The SMD for the before versus after supplementation HbA1C levels in the control group was -0.19 (95%CI: -0.45 - 0.07; I^2^ = 0.0%; P=0.159) (**Figure 3A**), while in the intervention group, it was -1.01 (95%CI: -1.55-(-0.46); I^2^ = 73.5%; P=0.000) (**Figure 3B**). We also compared the SMD between the treatment and control groups at both time points and found values of -0.14 (95%CI: -0.40 - 0.12; I^2^ = 0.0%; P=0.882) (**Figure 3C**) and -1.18 (95%CI: -1.83-(-0.54); I^2^ = 79.8%; P=0.000) (**Figure 3D**) for the before and after supplementation levels, respectively.

### 3.5 Effect of boswellia on lipid profile

We obtained data on the mean and SD of all lipid profile values (including TC, TG, LDL, and HDL) in the intervention and control groups before and after supplementation in all 5 included studies, except for the HDL reported in 4 out of 5 studies.

**Figure 4:**
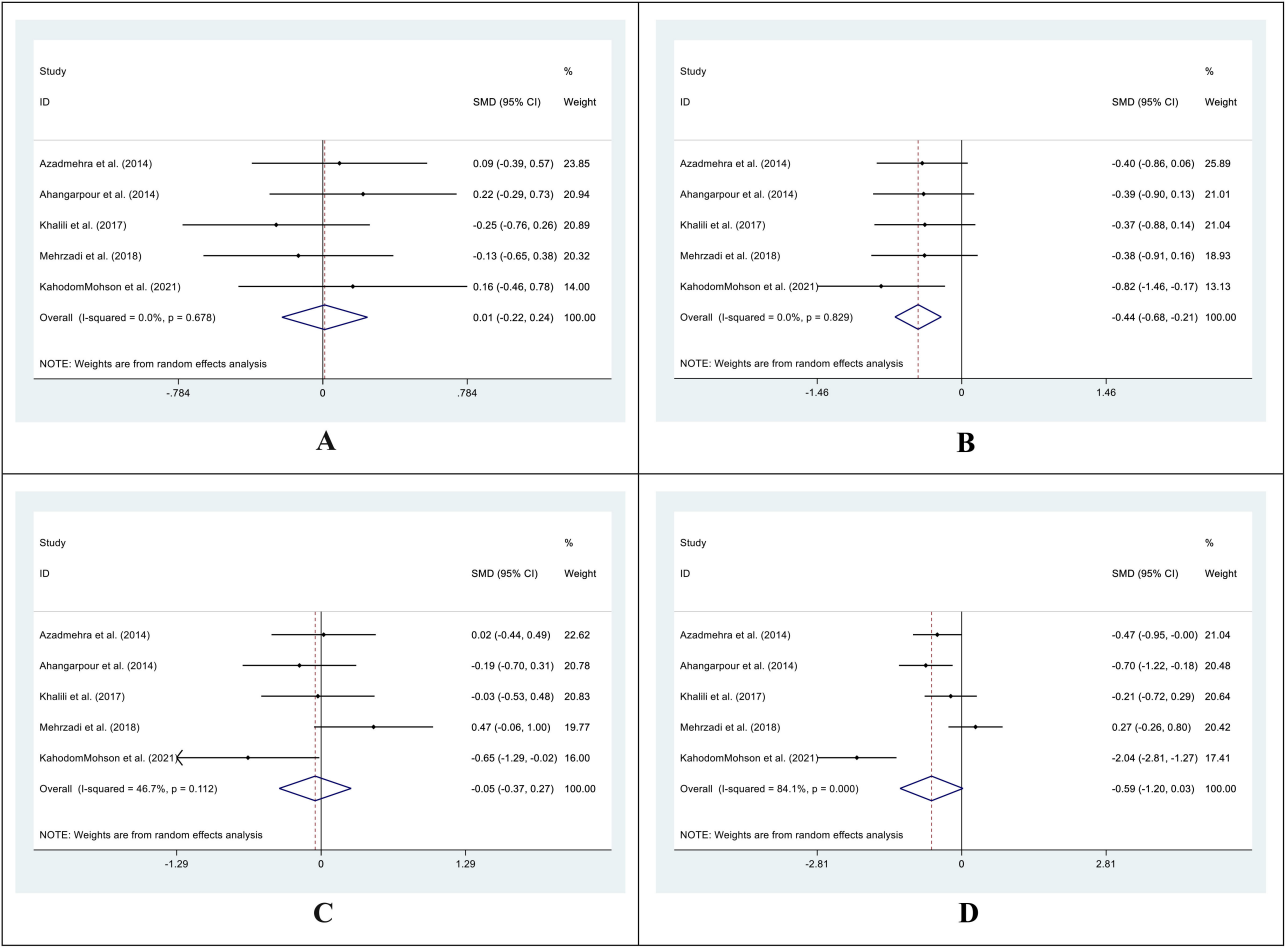
Forest plot of SMD for the TC. (**A**: before vs. after in the control groups; **B**: before vs. after in the intervention groups; **C**: intervention vs. control groups before supplementation; **D**: intervention vs. control groups after supplementation).

The SMD for the control group’s pre- vs. post-supplementation TC levels was 0.01 (95%CI: -0.22 - 0.24; I^2^ = 0.0%; P=0.930) (**Figure 4A**). In the intervention group, the SMD was -0.44 (95%CI: -0.68 – (-0.21); I^2^ = 0.0%; P=0.00) (**Figure 4B**). We also compared the SMD between the treatment and control groups at two different time points. The SMD for the pre-treatment level was -0.05 (95%CI: -0.37 to 0.27; I^2^ = 46.7%; P = 0.746) (**Figure 4C**), while for the post-treatment level, it was -0.59 (95%CI: -1.20 to 0.03; I^2^ = 84.1%; P = 0.060) (**Figure 4D**).

**Figure 5:**
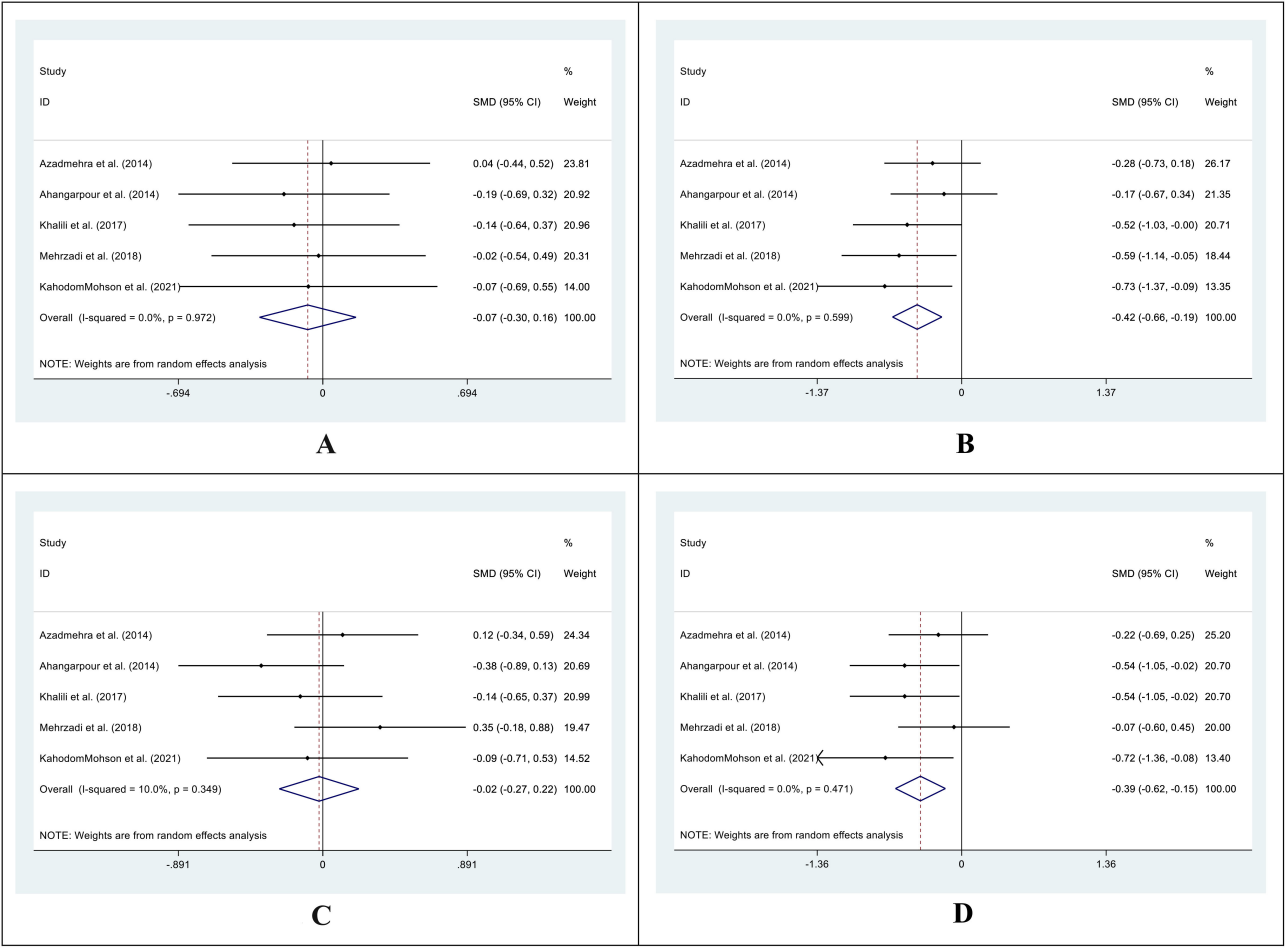
Forest plot of SMD for the TG. (**A**: before vs. after in the control groups; **B**: before vs. after in the intervention groups; **C**: intervention vs. control groups before supplementation; **D**: intervention vs. control groups after supplementation).

For TG levels, the SMD between the pre- and post-treatment in the control group was -0.07 (95%CI: -0.30-0.16; I^2^ = 0.0%; P=0.541) (**Figure 5A**). The SMD in the treatment group was -0.42 (95%CI: -0.66 – (-0.19); I^2^ = 0.0%; P=0.000) (**Figure 5B**). We analyzed the SMD between the treatment and control groups at two periods. The SMD for the pre-treatment level was -0.02 (95%CI: -0.27-0.22; I^2^ = 10.0%; P=0.854) (**Figure 5C**). For the post-treatment level, the SMD was -0.39 (95%CI: -0.62-(-0.15); I^2^ = 0.0%; P=0.001) (**Figure 5D**).

**Figure 6:**
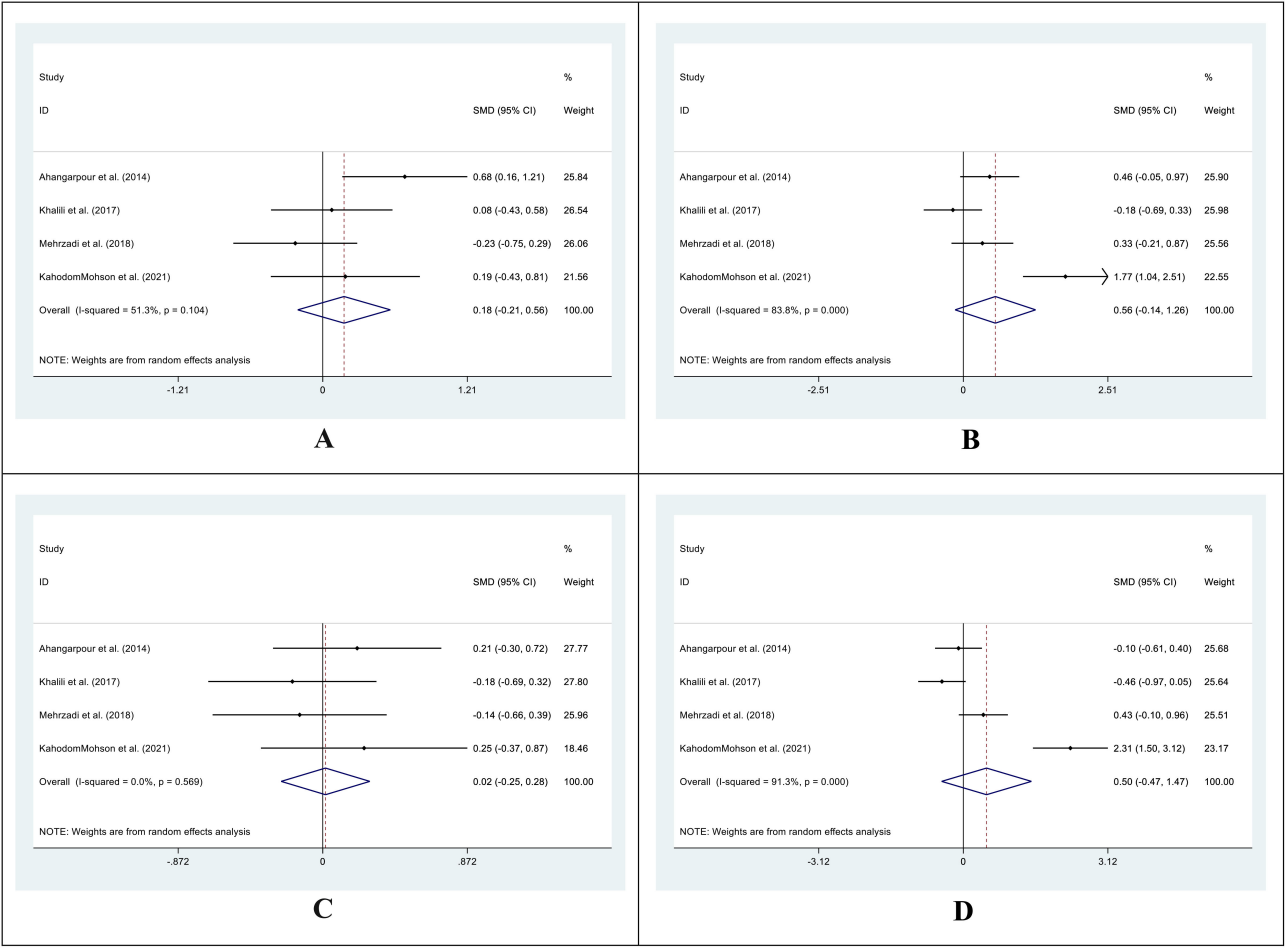
Forest plot of SMD for the HDL. (**A**: before vs. after in the control groups; **B**: before vs. after in the intervention groups; **C**: intervention vs. control groups before supplementation; **D**: intervention vs. control groups after supplementation).

The SMD in HDL levels between the pre- and post-treatment in the control group was 0.18 (95%CI: -0.21 -0.56; I^2^ = 51.3%; P=0.367) (**Figure 6A**), while in the treatment group was 0.56 (95%CI: -0.14 -1.26; I^2^ = 83.8%; P=0.118) (**Figure 6B**). We also compared the SMD between the treatment and control groups at two-time points. The SMD for the pre-treatment level was 0.02 (95%CI: -0.25 -0.28; I^2^ = 0.0%; P=0.903) (**Figure 6C**), while for the post-treatment level was 0.50 (95%CI: -0.47 -1.47; I^2^ = 91.3%; P=0.311) (**Figure 6D**).

**Figure 7:**
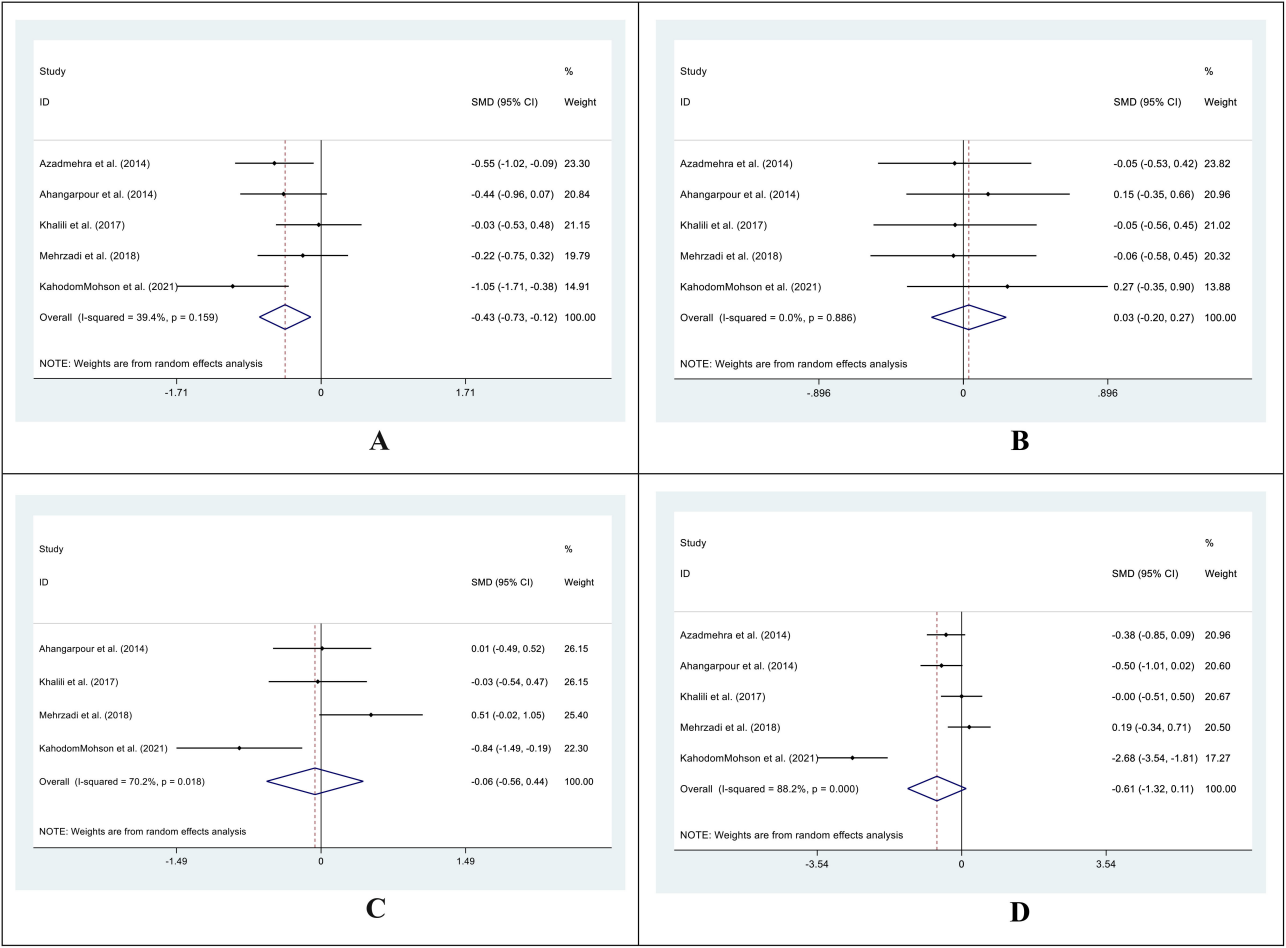
Forest plot of SMD for the LDL. (**A**: before vs. after in the control groups; **B**: before vs. after in the intervention groups; **C**: intervention vs. control groups before supplementation; **D**: intervention vs. control groups after supplementation).

In the treatment group, the SMD in LDL levels between pre- and post-treatment was -0.43 (95%CI: -0.73 – (-0.12); I^2^ = 39.4%; P=0.006) (**Figure 7A**), while in the control group, it was 0.03 (95%CI: -0.20 -0.27; I^2^ = 0.0%; P=0.775) (**Figure 7B**). Additionally, we compared the SMD at two different periods between the treatment and control groups. SMD was -0.06 (95%CI: -0.56 -0.44; I^2^ = 70.2%; P=0.805) (**Figure 7C**) for the pre-treatment level and -0.61 (95%CI: -1.32 -0.11; I^2^ = 88.2%, P=0. 0.097) (**Figure 7D**) for the post-treatment level.

### 3.6 Sub-group analysis and meta-regression

**Figure 8:**
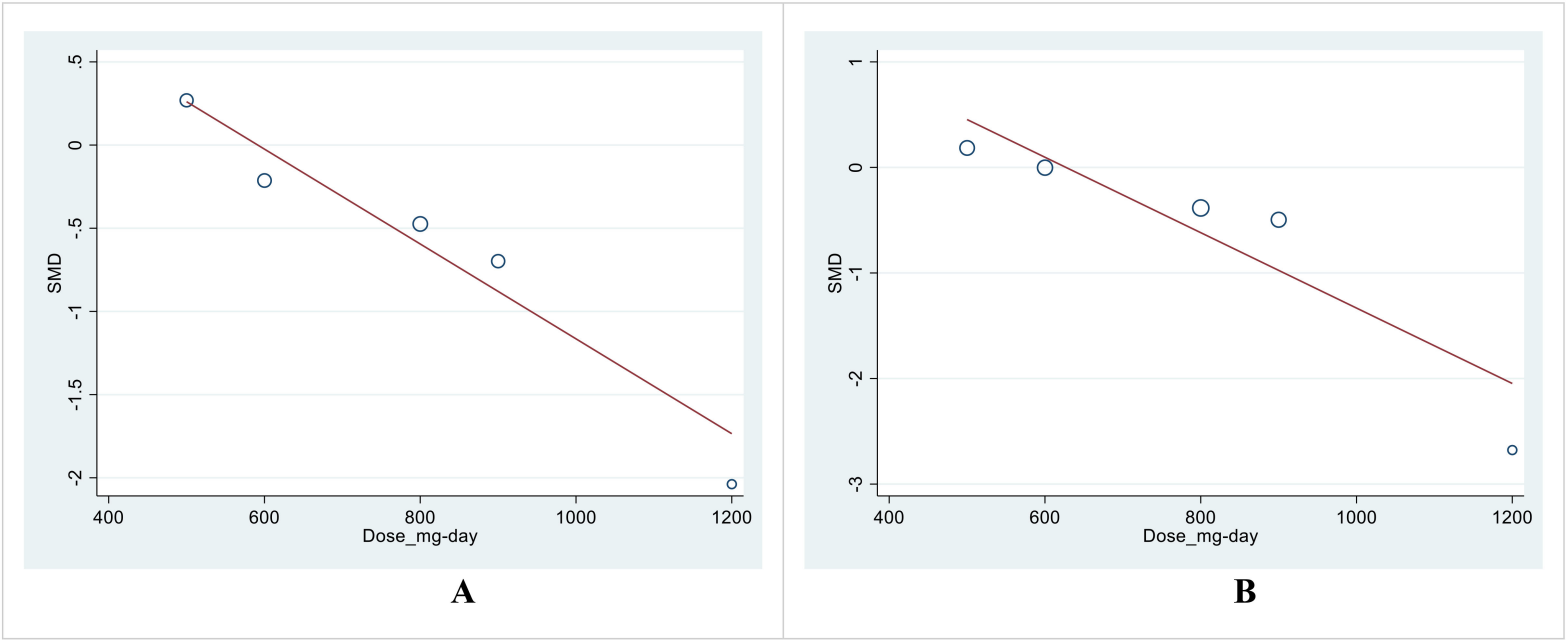
**(A)** Meta-regression analysis of SMD for the intervention and control in the post-treatment TC levels with a supplement dose. **(B)** Meta-regression analysis of SMD for the intervention and control in the post-treatment LDL levels with a supplement dose.

Due to the insufficient number of papers, we did not perform a subgroup analysis. However, we used meta-regression as an alternative to address heterogeneity. The meta-regression results for the effect size (SMD) with the dose and duration of treatment did not show significant results, except for the dose of therapy in post-treatment SMD between intervention and control groups in TC (P=0.041) and LDL (P=0.039) levels (**Table 5**; **Figures 8A, B**).

**Table 5:** Meta-regression.

Independent variable	Confounding variable	Comparison	P-value
***FBG***	Duration	After vs. Before	0.590
		Intervention vs. Control	0.615
	Dose	After vs. Before	0.144
		Intervention vs. Control	0.169
***HbA1C***	Duration	After vs. Before	0.412
		Intervention vs. Control	0.173
	Dose	After vs. Before	0.789
		Intervention vs. Control	0.442
***Total cholesterol***	Duration	After vs. Before	0.789
		Intervention vs. Control	0.525
	Dose	After vs. Before	0.452
		Intervention vs. Control	0.041
***Triglycerides***	Duration	After vs. Before	0.596
		Intervention vs. Control	0.938
	Dose	After vs. Before	0.912
		Intervention vs. Control	0.342
***HDL***	Duration	After vs. Before	0.920
		Intervention vs. Control	0.769
	Dose	After vs. Before	0.338
		Intervention vs. Control	0.475
***LDL***	Duration	After vs. Before	0.870
		Intervention vs. Control	0.564
	Dose	After vs. Before	0.149
		Intervention vs. Control	0.038

FBG, Fasting Blood Glucose; HbA1C, hemoglobin A1C; HDL, High-Density Lipoprotein; LDL, Low-Density Lipoprotein.

### 3.7 Publication bias

**Figure 9:**
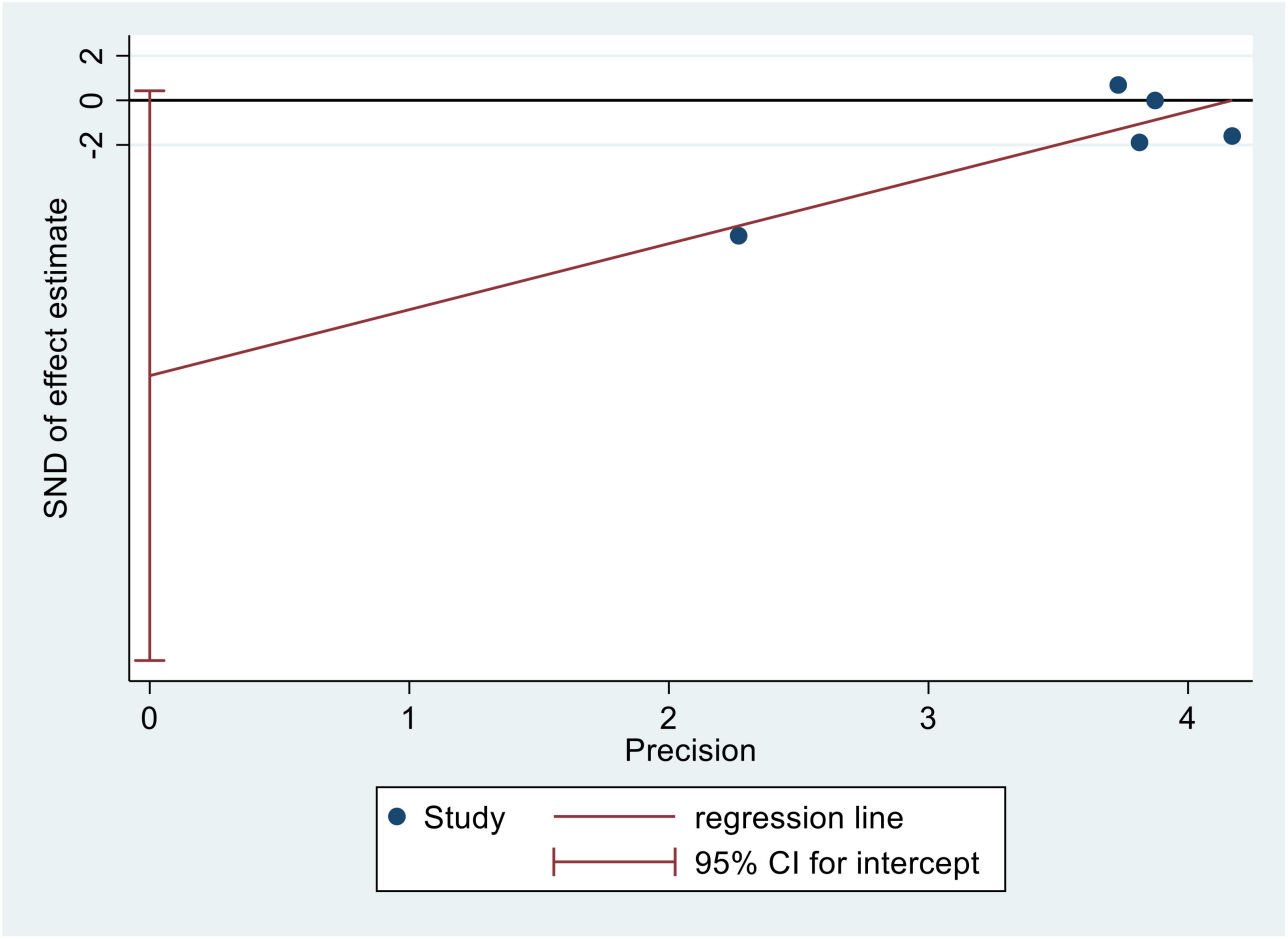
Egger’s publication bias plot for TC.

**Figure 9** displays Egger’s publication bias plot for TC. The analysis of this plot indicated the absence of publication bias (p=0.054), indicating that both positive and negative findings have been reported.

## 4 Discussion

To our knowledge, this study represents the first comprehensive systematic review and meta-analysis evaluating the effects of boswellia supplementation on the glycemic markers and lipid profile in patients with T2DM. The meta-analysis found that boswellia supplementation led to notable improvements in glycemic markers and lipid profiles. HbA1c levels showed significant reductions in the intervention group compared to the control while fasting blood glucose (FBG) improved but was not statistically significant. Additionally, there were significant decreases in total cholesterol (TC), triglycerides (TG), and LDL levels, with a slight but non-significant increase in HDL levels. These findings suggest that boswellia supplementation effectively enhances glycemic control and lipid profiles.

In the clinical practice of medicine, FBG and HbA1C are considered the backbone of DM diagnosis. FBG and HbA1C indicate the relative short-term and long-term glycemic control in the clinical evidence (25).

T2DM, as a multi-organ, metabolic, and chronic disease, is strongly associated with the development of dyslipidemia, contributing to life-threatening cardiovascular complications (26). It is essential to leverage anti-glycemic agents with a favorable impact on lipid metabolism, as they can provide optimal benefits in managing glycemic markers while simultaneously improving lipid indices (27).

Patients diagnosed with non-communicable diseases often experience multimorbidity, polypharmacy, and adverse drug reactions, leading to numerous complications (28). Regarding this matter, the trend toward utilizing natural phytonutrients in managing non-communicable diseases, especially T2DM, has emerged as an era of research interest. Since immemorial, complementary herbal medicine and phytonutrients have attracted considerable attention and yielded promising therapeutic outcomes (29).

Emerging evidence has shown that *Boswellia serrata*, a potential traditional herbal medicine, possesses favorable anti-inflammatory (30), antitumor (31), antimicrobial (32), and hepatoprotective effects (12). Surprisingly, preclinical research projects determined the antihyperglycemic and anti-hyperlipidemic properties of boswellia in a diabetic rat model induced by streptozotocin (33).

*Boswellia serrata*, commonly known as Indian frankincense, is a traditional medicinal plant used in Ayurveda since ancient times due to its anti-inflammatory and therapeutic properties. Its active components, particularly boswellia acids, have been reported to offer potential benefits by altering pathways leading to diabetes and related complications via inflammation (17–20).

Evidence has shown that boswellia has a significant regulatory effect on glycemic and lipid metabolism. It achieves this by protecting pancreatic beta cells and playing a crucial role in regulating insulin signaling, which is key in diabetes management. Additionally, it influences gluconeogenesis (34–36).

Pro-inflammatory cytokines play a crucial role in insulin resistance and T2DM development (37). In this regard, numerous studies demonstrated that the protective effect of boswellic acid on diabetes management could be explained by a significant improvement in the concentration of inflammatory markers, possibly via downregulating the Nuclear Kappa B (NF-kB) signaling pathways (38, 39). Moreover, the anti-oxidant effect of boswellia species can modulate the serum lipid level by reducing the Tumor Necrosis Factor α (TNF-α) and Interleukin-1β levels while increasing the adiponectin level (40). In this connection, Pandey et al. demonstrated that the boswellia extracts showed a remarkable decline in TC and a notable increase in HDL in the rat models (41). Interestingly, a computational analysis by Khan A. et al. revealed that boswellia acid extracts might potentially treat diabetes by inhibiting dipeptidyl peptidase-4. Their biochemical analysis suggested that the insulin receptors could be the therapeutic targets of boswellia acid extracts (42).

According to the previously determined favorable effect of boswellia in diabetic animal models, several clinical trials evaluated the antihyperglycemic and anti-hyperlipidemic effects of different doses of boswellia compounds in patients with T2DM (12–16). It is noteworthy to consider some points when interpreting the results of included studies. Mehrzadi et al. showed that eight weeks of treatment with 500 mg boswellia gum resin in T2DM patients did not significantly improve the glycemic and lipid profile compared with the placebo group (15). Another randomized clinical trial found that 1200 mg of boswellia powder for 12 weeks with or without a metformin supplement benefited T2DM patients with a significant reduction in blood glucose and HbA1C (16).

It is important to note that the varying results from the studies may be due to differences in factors such as sample size, dose, duration, mode of intervention, and measurement kit errors. Other confounding factors could impact the glycemic index results, which are worth discussing. Diabetic patients participating in trials may receive oral hypoglycemic agents, which may be a source of heterogeneity based on the particular type of anti-diabetic medications. Physical activity, lifestyle modification, weight loss, and supplementation treatment can significantly induce a lower blood glucose level compared to the alone supplementation therapy. Another important note is that boswellia compounds utilized in different studies may have different percentages of boswellia or other promising nutrients. Importantly, not all included studies used a specific form of boswellia compound, which should be considered. For example, Khalili et al. (14) performed a randomized clinical trial that showed how a mix of herbal compounds (olibanum, silymarin, and nettle) could lower blood glucose in people with T2DM for three months (14). Investigators should consider that concurrent phytonutrients or drugs may cause bias and distorted results during the research project.

The present systematic review and meta-analysis have some limitations. More importantly, our study includes a limited number of eligible studies with a small sample size. The included articles were heterogeneous, concerning the different characteristics of the population, as well as the diverse dose, quality, and duration of intervention. The short follow-up periods in studies limit the capability to evaluate the safety and long-term efficacy of boswellia on glycemic and lipid markers. Besides, most of the recruited studies were conducted in Iran, resulting in a limited generalization of our findings.

Furthermore, due to the limited number of included studies, our analysis needed to incorporate subgroup analysis. To address the heterogeneity precisely, we performed a meta-regression analysis. We observed a significant impact of intervention dose on the effect size for TC and LDL levels, providing a remarkable dose-response relationship in these outcomes. Correspondingly, our systematic review and meta-analysis necessitate the performance of large-scale, well-designed clinical trials with prolonged duration of intervention alone with long-term follow-up to elucidate further the potential therapeutic effect of boswellia on glucose control, lipid profile, safety, and tolerability in T2DM patients. The clinical implication of boswellia in managing glycemic and lipid profiles is crucial in preventing and managing cardiovascular complications in diabetic patients.

## 5 Conclusion

In conclusion, boswellia and its extract supplementation may positively impact glycemic markers and lipid profiles in patients with T2DM. However, well-designed clinical trials on a larger scale are recommended to validate these findings and fully understand their effects, with extended intervention durations and long-term follow-up. These future studies should aim to confirm the benefits, assess the sustainability of the effects, and explore any potential long-term outcomes or risks associated with boswellia supplementation.

## Data Availability

The original contributions presented in the study are included in the article/**Supplementary Material**. Further inquiries can be directed to the corresponding author.
