# Supplementary material for: Effect of boswellia (Boswellia serrata L.) supplementation on glycemic markers and lipid profile in type 2 diabetic patients: a systematic review and meta-analysis
Source: Front Clin Diabetes Healthc. 2024 Oct 10;5:1466408. doi: 10.3389/fcdhc.2024.1466408 (PMC11499236; doi:10.3389/fcdhc.2024.1466408)
Supplement: Supplementary file 1 [file DataSheet1.docx]

**Effects of Boswellia (*Boswellia serrata L.*) Supplementation on Glycemic Markers and Lipid Profile in Type 2 Diabetic Patients: A Systematic Review and Meta-Analysis**

**Supplementary materials**

**FBS
Control: Before vs. After**

**FBS. Int: Before vs. After**

**FBS. Cont: Before vs. After**

**FBS. Int: Before vs. After**

**HbA1C. Cont.: before vs. after**

**HbA1C. Int.: before vs. after**

**HbA1C int vs cont before**

**HbA1C int vs cont after**

**TC: Cont before vs. after**

**TC: int before vs. after**

**TC: before int vs cont**

**TC: after int vs cont**

**TG cont after vs before**

**TG int after vs before**

**TG before int vs cont**

**TG after int vs cont**

**HDL before int vs cont**

**HDL after int vs cont**

**HDL cont after vs before**

**HDL int after vs before**

**LDL before int vs. cont**

**LDL after int vs cont**

**LDL. Control group: Before vs. After**

**LDL. Intervention group: Before vs. After**

**Meta-regression of FBS**

**Meta-regression of HbA1C**

**Meta-regression of TC**

**Meta-regression of TG**

**Meta-regression of HDL**

**Meta-regression of LDL**
